# Evaluation of MODS and survival outcome in infectious diseases using SOFA and SAPS scores

**DOI:** 10.6026/973206300212262

**Published:** 2025-07-31

**Authors:** Kush Narayan Ghanghoria, Jha R.K, Sachin Parmar

**Affiliations:** 1 Department of General Medicine, Sri Aurobindo Institute of Medical Sciences (SAIMS), Indore, Madhya Pradesh, India; 2 Department of Community Medicine, V.K.S. Govt. Medical College, Neemuch, Madhya Pradesh, India

**Keywords:** Mortality prediction, SAPS, SOFA, Multiorgan dysfunction syndrome, ICU

## Abstract

The predictive ability of the Simplified Acute Physiology Score (SAPS) and Sequential Organ Failure Assessment (SOFA) in forecasting mortality in critically ill patients with infectious diseases leading to multiorgan dysfunction syndrome (MODS) is of interest. Hence, a total of 60 patients were analyzed. Data shows that SOFA outperformed SAPS in predicting mortality, with higher sensitivity, specificity, and area under the curve (AUC). Thus, SOFA is a more reliable tool for assessing mortality risk in ICU patients, improving clinical decision-making.

## Background:

Multiple organ dysfunction syndrome (MODS) represents one of the most challenging clinical scenarios in modern critical care medicine, characterized by the progressive and potentially reversible dysfunction of two or more organ systems in critically ill patients [1, 2]. This complex syndrome has emerged as a leading cause of morbidity and mortality in intensive care units globally, with mortality rates reaching as high as 82.6% when four or more organ systems fail simultaneously [3, 4]. The pathophysiology of MODS involves a dysregulated host response characterized by excessive pro-inflammatory mediator release, microvascular dysfunction, cellular metabolic reprogramming and mitochondrial damage, ultimately leading to organ failure and death [2, 5 and 6].Infectious diseases, particularly sepsis, constitute the predominant underlying etiology of MODS, accounting for approximately 28.7% of cases in critically ill patients [3, 4]. The clinical progression typically begins with infection-induced systemic inflammatory response syndrome (SIRS), which can rapidly evolve into sepsis and subsequently progress to septic shock and MODS [7, 8]. The relationship between sepsis and MODS is bidirectional and complex, with sepsis-induced MODS representing a virulent syndrome where mortality is directly proportional to the number of failing organ systems [7, 9]. Contemporary research indicates that sepsis-associated mortality is highly correlated with the development of MODS, with current studies reporting mortality rates ranging from 4.2% in mild organ dysfunction to 95.2% in severe cases [10, 11]. The accurate assessment and prediction of outcomes in patients with infectious disease-related MODS remains a critical clinical challenge, necessitating the development and validation of reliable scoring systems. Among the various prognostic tools available, the Sequential Organ Failure Assessment (SOFA) and Simplified Acute Physiology Score (SAPS) have emerged as the most widely utilized and validated systems in critical care settings [10, 11, 12-13].

The SOFA score, originally designed as the Sepsis-related Organ Failure Assessment, evaluates dysfunction across six organ systems: respiratory, cardiovascular, hepatic, coagulation, renal, and neurological, with scores ranging from 0 to 24 points [3, 4]. Its simplicity and dynamic nature allow for daily monitoring of disease progression and response to treatment, making it particularly valuable in infectious disease contexts [10, 13]. The SAPS scoring system, designed to provide mortality risk assessment based on physiological derangements within the first 24 hours of ICU admission, offers a different approach to prognostication [12, 14]. While SAPS incorporates age, admission type, and underlying diseases alongside physiological parameters, its static nature contrasts with the dynamic assessment capabilities of SOFA [13]. Recent comparative studies have demonstrated varying performance characteristics between these scoring systems, with some investigations suggesting SOFA's superiority in infectious disease populations due to its ability to capture organ-specific dysfunction patterns [10, 15]. The clinical significance of accurate prognostication in infectious disease-related MODS extends beyond mortality prediction to include resource allocation, treatment intensity decisions, and family counseling [16]. Contemporary research has shown that SOFA scores of 0-1 are associated with 100% survival, while scores exceeding 11 correlate with 100% mortality in certain populations [11]. Similarly, SAPS scores have demonstrated significant predictive value, with studies reporting area under the receiver operating characteristic curve (AUROC) values ranging from 0.742 to 0.855 for mortality prediction [10, 15]. Understanding the comparative performance of SOFA and SAPS scoring systems in infectious disease populations is crucial for optimizing clinical decision-making and improving patient outcomes in the era of precision medicine. Therefore, it is of interest to evaluate MODS and survival outcome using SOFA and SAPS scores.

## Materials and Methods:

## Study design:

The present study is an observational study aimed at evaluating the mortality prediction in critically ill patients using the SAPS (Simplified Acute Physiology Score) and SOFA (Sequential Organ Failure Assessment) scores in patients with infectious diseases leading to multiorgan dysfunction syndrome (MODS) in an Intensive Care Unit (ICU) setting.

## Study setup:

The study was conducted in the Department of General Medicine, Sri Aurobindo Medical College and Post Graduate Institute, Indore (Madhya Pradesh), India. The hospital serves as a tertiary care center with a dedicated ICU for critically ill patients.

## Study duration:

The duration of the study was 18 months, from December 2016 to May 2018. The study included both male and female patients diagnosed with infectious diseases leading to MODS, who were admitted to the ICU during the study period.

## Study population:

Patients diagnosed with infectious diseases resulting in multiorgan dysfunction syndrome, and who were admitted to the ICU of Sri Aurobindo Medical College and Post Graduate Institute and seeking medical attention during the study period, were included in this study. Informed written consent was obtained from all patients or their legal attendants for participation.

## Inclusion criteria:

[1] Age range: 18 to 90 years (both male and female).

[2] Condition: Patients diagnosed with infectious diseases leading to multiorgan dysfunction syndrome requiring admission to the ICU.

[3] Consent: All patients or their attendants who provided written informed consent for participation in the study.

## Exclusion criteria:

[1] Patients below 18 years of age.

[2] Patients with non-infectious causes of multiorgan dysfunction.

[3] Patients who refused to participate in the study.

## Sample size:

The sample size for this study was estimated to be 60 patients based on medical records and feasibility criteria. This sample size was considered adequate based on prior studies and institutional records to achieve meaningful statistical results.

## Procedure:

Upon admission to the ICU, all patients were thoroughly assessed. The following steps were undertaken:

[1] Medical History: A detailed medical history including personal, medical, and surgical history was taken from each patient.

[2] Clinical Examination: Comprehensive clinical examination was performed by trained physicians to assess the severity of the illness.

[3] Investigations: All patients underwent a series of relevant investigations to assess their physiological and biochemical status:

1) Complete Blood Count (CBC)

2) Total Bilirubin

3) Serum Creatinine

4) Serum Urea

5) Arterial Blood Gas (ABG) Analysis

6) Serum Electrolytes

These investigations were essential for the calculation of SAPS and SOFA scores. Data from blood investigations were transcribed directly from the laboratory reports into a specially designed prestructuredproforma.

## Data collection and Methods:

A prestructuredproforma was designed for systematic data collection. The proforma captured patient demographics, medical history, clinical examination findings, and results from biochemical tests and investigations. The primary outcome measure was the survival status of patients at the time of ICU discharge.

[1] SAPS and SOFA Scoring: Both SAPS and SOFA scores were calculated for each patient. The initial SAPS and SOFA scores were based on the worst values recorded during the first 24 hours of ICU admission.

[2] Outcome: The primary endpoint of the study was the survival status (survived or died) at the time of ICU discharge.

## Statistical analysis plan:

[1] Descriptive statistics will be used to summarize the demographic and clinical characteristics of the study population.

[2] SAPS and SOFA Scores will be compared between survivors and non-survivors using Independent t-test.

[3] ROC curve analysis will be employed to assess the discriminatory ability of the SAPS and SOFA scores for predicting mortality.

[4] A p-value < 0.05 will be considered statistically significant.

The statistical software used for analysis will include tools like SPSS (Statistical Package for the Social Sciences) or R for generating the relevant statistical results.

## Ethical considerations:

This study was conducted in compliance with the ethical guidelines laid down by the institutional review board of Sri Aurobindo Medical College and Post Graduate Institute. Informed written consent was obtained from all study participants, and confidentiality of patient data was maintained throughout the study.

## Results:

Table 1 (see PDF) presents a comparison of age, SAPS, and SOFA scores between survivors and non-survivors. The mean SAPS and SOFA scores were significantly higher in patients who died compared to those who survived (P = 0.000 for both). However, the age difference between the two groups was not statistically significant (P = 0.650). Table 2 (see PDF) & Figure 1 (see PDF) compares the performance of the SAPS and SOFA scores in predicting mortality using the area under the ROC curve (AUC). Both scores demonstrated excellent discriminatory ability with high AUC values. SOFA score (AUC = 0.967) was a better predictor of mortality than the SAPS score (AUC = 0.925), both with statistically significant P-values (P < 0.001). Table 3 (see PDF) & Figure 1 (see PDF) provides key coordinates from the ROC curve for SAPS and SOFA scores. The optimal cut-off value for SAPS was 50.50, with sensitivity of 84.6% and specificity of 85.1%. For SOFA, the optimal cut-off was 10.50, yielding perfect sensitivity (100%) and a high specificity of 93.6%. Table 4 (see PDF) presents the results of the independent t-test for age, SAPS, and SOFA scores. The SAPS and SOFA scores were significantly higher in the non-survivor group, with both tests having P-values of 0.000, indicating they are reliable mortality predictors. The age difference was not statistically significant. This Table 5 (see PDF) presents a comparison of age, SAPS, and SOFA scores between survivors and non-survivors. The mean SAPS and SOFA scores were significantly higher in patients who died compared to those who survived (P = 0.000 for both). However, the age difference between the two groups was not statistically significant (P = 0.650).This table compares the performance of the SAPS and SOFA scores in predicting mortality using the area under the ROC curve (AUC). Both scores demonstrated excellent discriminatory ability with high AUC values. SOFA score (AUC = 0.967) was a better predictor of mortality than the SAPS score (AUC = 0.925), both with statistically significant P-values (P < 0.001).This table provides key coordinates from the ROC curve for SAPS and SOFA scores. The optimal cut-off value for SAPS was 50.50, with sensitivity of 84.6% and specificity of 85.1%. For SOFA, the optimal cut-off was 10.50, yielding perfect sensitivity (100%) and a high specificity of 93.6%.This table presents the results of the independent t-test for age, SAPS, and SOFA scores. The SAPS and SOFA scores were significantly higher in the non-survivor group, with both tests having P-values of 0.000, indicating they are reliable mortality predictors. The age difference was not statistically significant.This table summarizes the average SAPS and SOFAT scores for survived and died patients. As seen, both SAPS and SOFA scores were considerably higher in the deceased group, highlighting their utility as strong indicators of mortality risk.

## Discussion:

The study findings demonstrating superior performance of SOFA over SAPS in mortality prediction (AUC = 0.967 vs 0.925) align strongly with multiple recent investigations. A comparative analysis by Morkar *et al.* found that while both SAPS II and SOFA showed no significant distinction between predictive capabilities from initial day (p=0.079) to fifth day (p=0.062), the SOFA score exhibited superior discriminatory performance in specific clinical contexts [17]. The excellent AUC values reported in the study (SOFA: 0.967, SAPS: 0.925) are consistent with findings from Basiri *et al.* who demonstrated that SOFA had significantly higher AUC (0.921) compared to SAPS III (0.855) and MPM II (0.839) in COVID-19 ICU patients. Their study confirmed SOFA's superior predictive accuracy with 92.52% sensitivity and 80.0% specificity [18]. The study finding that SOFA achieved perfect sensitivity (100%) and high specificity (93.6%) at a cut-off of 10.50 is supported by extensive literature. Kumar *et al.* demonstrated that SOFA score on day 3 provided the best mortality prediction with an AUC of 0.8104, outperforming APACHE II (0.7879) and APACHE IV in sepsis patients with multiple organ dysfunction syndromes [19]. Sequential organ failure assessment has consistently shown superior performance in infectious disease contexts. A study by Acharya *et al.* found that initial SOFA score was superior to SAPS not only as a mortality predictor but also as an indicator of multiple organ dysfunctions in infectious diseases, with SOFA demonstrating both sensitivity and specificity while SAPS lacked sensitivity [2]. The study's SAPS cut-off value of 50.50 yielding 84.6% sensitivity and 85.1% specificity is consistent with literature findings. However, multiple studies have highlighted SAPS limitations in certain populations. A comprehensive analysis by Aminiahidashti *et al.* found that APACHE II and SAPS II had similar value in predicting 1-month mortality, with AUC values of 0.72 and 0.75 respectively, but both demonstrated calibration limitations [20].

The age-related findings in the study, showing age alone was not sufficient for mortality prediction (P = 0.650), are strongly supported by recent research. A Malaysian ICU study found that while age was the strongest predictor for ICU mortality with elderly patients having hazard ratio of 4.777, disease severity scores consistently outperformed age alone in mortality prediction [21]. The study's ROC analysis demonstrating excellent discriminatory performance aligns with meta-analytical findings. A systematic review by Saleh *et al.* comparing multiple ICU scoring systems found that APACHE III showed the best discrimination followed by APACHE II and SOFA, with all scoring systems significantly associated with mortality (F = 62.772, p = 0.000) [22]. Recent machine learning studies have validated the superiority of dynamic scoring systems like SOFA over static systems like SAPS. Furqan *et al.* demonstrated that APACHE II had highest sensitivity (77.53%), specificity (94.28%) and accuracy (85.45%) compared to SAPS II (47.29% sensitivity, 87.32% specificity) and SOFA (73.37% sensitivity, 60.28% specificity) [23]. However, these findings emphasize the context-dependent nature of scoring system performance. The study's conclusion that SAPS and SOFA scores are more reliable indicators of mortality risk than age is supported by surgical outcome research. A large-scale study by Dunlop *et al.* found that severity of illness was a much better predictor of outcome than age (p < 0.001), recommending that age should not be used in isolation for clinical decisions [16]. However, recent research has shown age-related variations in scoring system performance. de Groot *et al.* demonstrated that disease severity scores performed poorly in older patients (AUC range 0.56-0.64) compared to younger patients (AUC range 0.72-0.86), suggesting that age-specific modifications may be necessary for optimal performance [24]. The study findings are further validated by recent time-dependent ROC analyses showing that SOFA maintains predictive capacity consistently over time while other scoring systems may deteriorate. Multi-center studies have confirmed that SOFA-based models are competitive with SAPS II models in predicting mortality in general medical and surgical ICU patients [25]. Machine learning applications have consistently demonstrated that traditional scoring systems remain relevant when enhanced with computational approaches. Recent studies show that XGBoost models using SOFA components achieve AUC values of 0.918, while maintaining clinical interpretability [26].

## Conclusion:

SAPS and SOFA scores are valuable tools for predicting mortality in critically ill patients. SOFA score, in particular, demonstrated superior predictive ability and should be prioritized in clinical practice for its accuracy in identifying high-risk patients. Although age does not appear to significantly influence mortality outcomes, the use of these scoring systems provides a more objective and evidence-based approach to patient management, potentially improving patient outcomes in intensive care settings.
